# The intervention of music education on adolescents’ subjective well-being from the perspective of sustainable development: a bibliometric review based on literature from 2000 to 2024

**DOI:** 10.3389/fpsyg.2025.1617097

**Published:** 2025-06-16

**Authors:** Lan Shen, Weijia Yang

**Affiliations:** ^1^College of Modern Music, Shandong University of Arts, Jinan, China; ^2^Institute of Future Education, Yonsei University, Seoul, Republic of Korea; ^3^Department of Global Music, Kyonggi University, Seoul, Republic of Korea

**Keywords:** music education, sustainable development, psychology, adolescent, well-being

## Abstract

With the United Nations’ 2030 Agenda elevating Good Health and Well-Being (SDG 3) and Quality Education (SDG 4) to global priorities, music education, by virtue of its cross-cultural character and emotional resonance, is increasingly recognized as a potential pathway for fostering adolescents’ sustainable development competencies. Seventy-six core publications were retrieved from the Web of Science, MEDLINE, and ProQuest databases, and bibliometric and knowledge-mapping analyses were conducted using CiteSpace and VOSviewer. The field’s trajectory was deconstructed along three dimensions: temporal (annual publication output and author contribution levels), spatial (national participation and institutional collaboration density), and content (high-frequency keyword clustering and evolution of emerging themes). Findings reveal a two-stage “hiatus–surge” pattern in publication trends; the emergence of a collaborative network among core authors, albeit with an imbalanced geographic distribution dominated by North America and Europe; and five principal thematic clusters following a three-stage spiral progression—from targeted education interventions for special groups, through general adolescent development, to professional public-health services. Through a sustainable development–oriented, multidimensional evaluation framework and a standardized literature-review paradigm, multifaceted mechanisms by which music education enhances adolescents’ subjective well-being are uncovered, and evidence-based recommendations for sustainable education reform are provided (PROSPERO Registration ID: CRD420251030162).

## 1 Introduction

In the current context of global educational transformation and the prominent issue of adolescent mental health, the concept of sustainable development is extending from the economic and social levels to the individual domain, aiming, through education, to cultivate “whole persons” equipped with lifelong capacities for well-being. Globally, approximately 30% of adolescents experience psychological problems such as depression and anxiety, and this statistic continues to rise (Anderson et al., 2025). The United Nations’ 2030 Agenda for Sustainable Development has incorporated “social–emotional competence cultivation” into the core indicators of SDG 4: Quality Education (Goyal et al., 2024). The 2024 UNESCO International Forum on the Future of Education held in Suwon, South Korea, further emphasized the strategic positioning of “nurturing global citizens with lifelong well-being capabilities” (Popa, 2024). It is therefore evident that human sustainable development requires empowerment and support from psychology through sustainable education to enhance individual psychological well-being.

The adolescent stage is a critical period for the consolidation of character strengths and the formation of lifelong developmental capacities, which hold significant importance for sustainable development. Active sustainable education can cultivate core strengths such as creativity, resilience, and empathy (Vazquez-Marin et al., 2023), and in psychology, subjective well-being (SWB) serves as an important reference for positive sustainable education (Du et al., 2024). Subjective well-being is defined as the integrated cognitive appraisal and affective experience of one’s quality of life (Kahneman and Krueger, 2006), and its core components (life satisfaction, positive affect, and negative affect regulation) not only serve as key indicators for assessing mental health but also correspond to the United Nations Sustainable Development Goals of Good Health and Well-Being (SDG 3) and Quality Education (SDG 4). The former emphasizes the cultivation of social–emotional competencies required for “whole-person development” through education (Sweileh, 2020), while the latter incorporates psychological well-being as a central dimension of human health (Unterhalter, 2019). From a psychological perspective, the enhancement of subjective well-being relies not only on immediate affective experiences but also on systematic educational interventions designed to build enduring psychological support (Witter et al., 1984).

Music education serves as the key bridge linking short-term emotional pleasure with lifelong well-being capacities, thereby promoting the sustainable development of adolescents’ subjective well-being. As an educational form integrating esthetic experience, emotional expression, and social interaction, its impact on adolescents’ subjective well-being has emerged as a cross-disciplinary focus in educational psychology and arts education research (Papinczak et al., 2015). Music stimuli activate the prefrontal cortex and limbic system reward circuits (Blum et al., 2010), leading to elevated secretion of dopamine and serotonin (Chanda and Levitin, 2013), and enabling immediate emotional regulation and positive affect induction. A positive correlation exists between music education and cognitive regulation in college students (Wang et al., 2022), and sustained choir training has been shown to significantly enhance adolescents’ sense of social collective identity (Barrett and Zhukov, 2023). Thus, music education provides psychological support for the long-term maintenance of adolescents’ subjective well-being through multiple pathways, including neurophysiological activation and the construction of social skills.

From the perspective of sustainable development, research on the topic of “the impact of music education on adolescents’ subjective well-being” remains limited, with systematic investigation into this theme being particularly scarce. To address these gaps, the purpose of this study is to conduct a systematic literature review to provide quantitative analysis and qualitative description of the overall research status, emerging hotspots, and evolutionary trends, achieving a panoramic scan and in-depth deconstruction of the field’s current landscape. The study also attempts to discuss the causal mechanisms of “music education intervention-adolescent development-well-being enhancement,” aiming to provide evidence-based intervention strategies for global education policymakers. This research seeks to construct a three-dimensional analytical framework of “time–space-content” to achieve these objectives:

(1)  RQ1: In the temporal dimension, what trends characterize the annual publication output in this field? Has a stable corpus of source journals been established?
(2)  RQ2: In the spatial dimension, how are core authors distributed, and what characteristics distinguish different countries’ research contributions?
(3)  RQ3: In the content dimension, what are the frontier topics, and how have these hotspots evolved over time?

## 2 Methods

### 2.1 Research methodology

Systematic literature review, as the core methodology of scientific research, offers value by ensuring rigor in literature screening, controllability of research quality, and reproducibility of findings through standardized operational procedures (Yang et al., 2024b). For the research topic examining the impact of music education on adolescents’ subjective well-being from the sustainable education perspective, the PICO framework (Schiavenato and Chu, 2021) was applied to traverse Population (P), Intervention (I), Comparison (C), and Outcome (O) within search strings, and an interdisciplinary database search strategy was adopted to ensure comprehensive literature collection. Stringent adherence to the PRISMA (Preferred Reporting Items for Systematic Reviews and Meta-Analyses) guidelines (Page et al., 2021) facilitated transparent literature selection via a multilevel screening process. In the data-processing phase, bibliometric tools including Zotero, VOSviewer, and CiteSpace (Pan et al., 2018) were employed to achieve in-depth deconstruction of research data (Pan et al., 2018).

“Music education,” as the core research subject, encompasses social functions such as esthetic cultivation, cognitive development, and motivational enhancement. This study focuses on its interventional perspective on subjective well-being from the lens of music therapy—namely, through systematic musical practices (e.g., rhythm perception training, improvisational music creation, collective choral experiences), it aims to purposefully influence individual psychological regulation, cognitive capability development, and social behavior shaping. This research approach, which integrates the mechanisms of music learning in educational settings with the logic of psychological intervention in therapeutic contexts, fundamentally constitutes an interdisciplinary exploration in the field of music psychology.

The scientific rigor of the methodological framework derives from its precise alignment with the research questions. Rigorous application of the PICO framework ensured highly targeted literature retrieval; the PRISMA screening process guaranteed the scientific validity and relevance of included studies; and integration of multiple bibliometric tools enabled a multidimensional deconstruction of this complex, interdisciplinary field. The framework not only underpins investigation of the current status and evolutionary patterns of the “Sustainable Development—Music Education—Adolescent Well-Being” association mechanisms but also establishes a replicable scientific paradigm for subsequent educational intervention research and policy translation. Pre-registration in PROSPERO was completed under ID CRD420251030162.

### 2.2 Search criteria

To ensure the quality of the included data, this study adopted a scientifically validated search strategy based on multiple prior studies and strictly adhered to PRISMA guidelines. Web of Science (WOS), ProQuest (PQ), and MEDLINE were selected as the core databases for literature retrieval. These databases encompass disciplines such as psychology, musicology, and education, and their indexed core journal collections provide a high-quality benchmark for literature screening. The time span was set from January 1, 2000, to December 31, 2024, to ensure both chronological coverage and disciplinary diversity.

During the literature search phase, a multi-layered keyword combination strategy was designed based on the PICO framework. The four thematic keywords—“music,” “education,” “adolescent,” and “subjective well-being”—formed the foundation of the search. Given the diversity of keyword variations, the study also incorporated alternative keyword formulations. The research population is “adolescents aged 6 to 18.” Considering variations in school systems across countries, the search terms “middle school student*” and “junior high school student*” are used to focus on data for adolescents aged 12 to 14, thereby avoiding omission of the target population without limiting the search coverage. As shown in Table 1, derivative terms were expanded under each PICO dimension. For instance, in the case of Web of Science, the specific search query employed was:

**Table 1:** The PICO framework was used for the search.

Terms	Meaning	Derivative scope
Population (P)	Adolescents aged 6 to 18.	Refers to individuals receiving education from primary through secondary levels.
Intervention (I)	Music education practices.	Encompasses structured classroom music instruction, extracurricular musical activities, and long-term instrumental learning.
Comparison (C)	General education settings without music education.	Control groups in which the curriculum does not include regular or systematic music instruction.
Outcome (O)	Multidimensional assessment of adolescent mental health.	Includes subjective well-being indicators such as life satisfaction, positive affect, social well-being, and psychological resilience.

TS = ((“music*”) AND (“education” OR “pedagogy” OR “learning” OR “training”) AND (“adolescent*” OR “teen*” OR “youth*” OR “middle school student*” OR “junior high school student*”) AND (“life satisfaction” OR “quality of life” OR “subjective well-being” OR well-being OR “social well-being”)).

### 2.3 PRISMA screening

**Figure 1:**
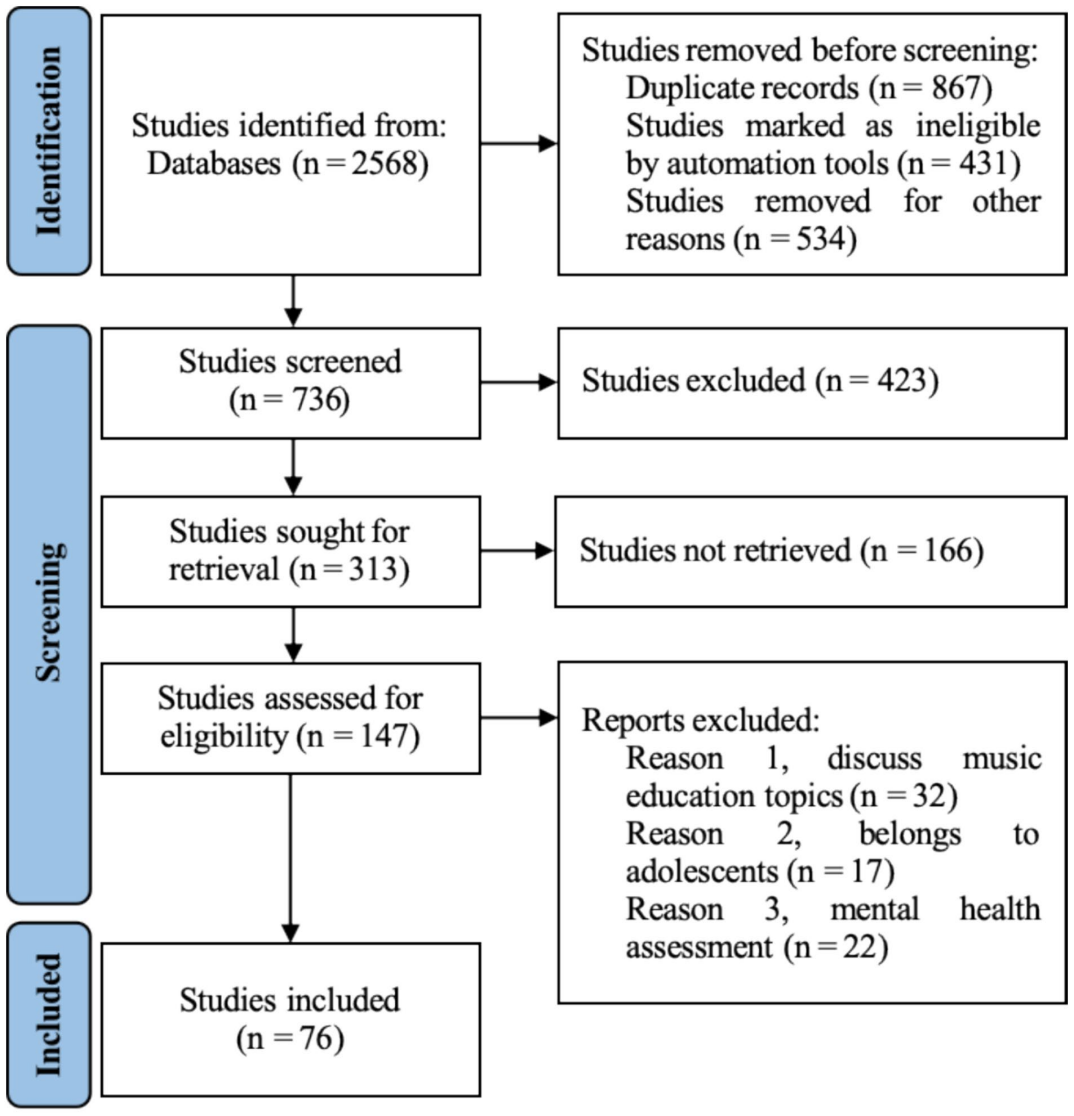
PRISMA article screening methodology.

To ensure precision and high quality of the literature data, PRISMA guidelines were strictly followed, and Zotero reference management software was used to perform a four-phase, stepwise screening of the 2,568 records initially retrieved from four databases (see Figure 1).

(1)  Stage 1: Data preprocessing was performed by removing 867 duplicate records via software de-duplication and excluding 431 retracted publications due to quality concerns. To ensure the reliability of the bibliometric analysis, 534 book chapters without DOIs were further excluded. In total, 1,832 records were removed, leaving 736 for the next stage.
(2)  Stage 2: Title-and-keyword screening for weak relevance was conducted. Independent evaluation by two reviewers, using a “single-agreement retention” principle, led to the exclusion of 423 records unrelated to the topic, with 313 advancing to validity verification.
(3)  Stage 3: Record completeness was verified through DOI matching and cross-database retrieval. A total of 166 records lacking DOIs and without accessible preprints were excluded, resulting in 147 records entering in-depth screening.
(4)  Stage 4: Abstract and full-text screening for strong relevance was undertaken. Two independent reviewers conducted detailed readings against three inclusion criteria—discussion of music education, focus on adolescent populations, and involvement of psychological health assessment—with domain experts consulted on disputed records. Ultimately, 71 records were excluded, yielding 76 high-quality studies as the final sample, thereby ensuring that all included records closely aligned with the research topic.

### 2.4 Structural coding

A three-tier coding framework was constructed to achieve systematic mapping of article content, research questions, and analytical findings. Upon completion of the screening process, the 76 included studies were subjected to structured coding, with three categories of core metadata collected (see Table 2). Two researchers independently applied the predefined coding scheme to annotate the initially screened articles, using each study’s basic bibliographic information as the classification criterion.

**Table 2:** Code framework.

Category	Code	Description
RQ1 (Section 3.1: Time)	Database	WOS / PQ / MEDLINE
	Research type	Article / Review / Books / Dissertation Thesis / Clinical Trial
	Year	Year of publication
RQ2 (Section 3.2: Space)	Authors	Important authors and team
	Institution	The organization to which the author group belongs
	Countries	Global research geography
RQ3 (Section 3.3: Content)	Research topics	Clusters of the main arguments of the paper
	Frontier hotspots	The sudden hot words studied in recent years
	Evolution timeline	The main threads of research and development

Data coding employed a combination of open coding and consensus decision-making, following the reliability testing framework of Gaur and Kumar (2018). Two researchers independently completed the coding. Initial results indicated full agreement on 73 studies. Coding consistency was calculated using the following formula (Equations 1, 2):


(1) $$\text{Average mutual agreement}:K=\frac{M}{N}\times 100\%$$

(2) $$\text{Reliability}:R=\frac{N\times K}{1+(n-1)\times K}$$

Given *M* = 73 and *N* = 76, substitution into the consistency formula yields a Cohen’s *κ* of 0.96 and an inter-rater reliability R of 0.98, indicating that the coding framework possesses high reliability and thus provides a solid methodological foundation for subsequent quantitative statistical analyses and qualitative theoretical interpretations. To ensure research transparency, the metadata for all 76 journal articles have been made publicly available on the Mendeley Data platform (Yang, 2025).

## 3 Results

### 3.1 Time analysis (RQ1)

#### 3.1.1 Metadata statistics of databases and research types

After standardizing the data for the 76 included publications, a multidimensional classification and statistical summary was produced (see Table 3). In terms of source database distribution, WOS indexed 44 publications (57.9%), PQ indexed 25 publications (32.9%), and MEDLINE indexed 7 publications (9.2%), indicating that research outputs are primarily concentrated in core databases of education and psychological sciences. Regarding research type distribution, Articles accounted for 40 publications (52.6%), reflecting the predominance of empirical studies; Dissertation Theses comprised 25 publications (32.9%), offering extensive foundational theoretical exploration; Reviews totaled 9 publications (11.8%), systematically mapping the research landscape; and one Evaluation Study and one Case Report were also included, demonstrating diversity in research formats. All data were uniformly coded to form a structured dataset, providing a standardized basis for subsequent bibliometric analyses.

**Table 3:** Data abstracts.

Category		Quantity
Sum	Publications	76
Database	WOS	44
	PQ	25
	MEDLINE	7
Research type	Article	40
	Dissertation Thesis	25
	Review	9
	Evaluation Study	1
	Case Reports	1

#### 3.1.2 Year distribution statistics

**Figure 2:**
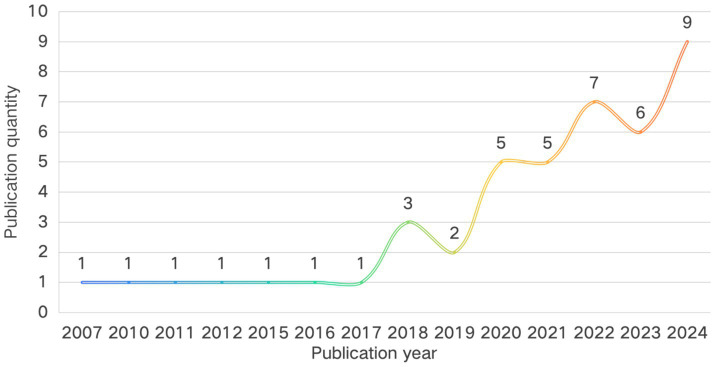
Time distribution of publications.

Using annual publication volume as a quantitative indicator of field development, bibliometric analysis reveals the temporal evolution trajectory of this field (Figure 2). A two-stage “hiatus–surge” pattern emerges. Prior to 2018, the field remained in its theoretical infancy, with a cumulative total of seven publications over 17 years (an average of 0.4 per year) and multiple publication gaps (e.g., no related outputs during 2000–2007, 2008–2009, and 2013–2014), suggesting that the intersection of music education and adolescent well-being from a sustainable education perspective had not yet attracted scholarly attention. The year 2018 marked a developmental turning point, with annual output exceeding two publications for the first time (three publications). Following 2020, the field entered an exponential growth phase, accumulating 32 publications between 2020 and 2024—42 percent of the total—with nine publications in 2024, representing a 200 percent increase over 2018. This trend aligns temporally with the advancement of the global Sustainable Development Goals and the rising focus of educational psychology on positive development indicators, indicating a shift from concept-introduction to a flourishing empirical research phase. With the continued integration of interdisciplinary methods and greater policy and practice demands, research activity is expected to remain robust.

### 3.2 Space analysis (RQ2)

#### 3.2.1 Author collaboration network

**Figure 3:**
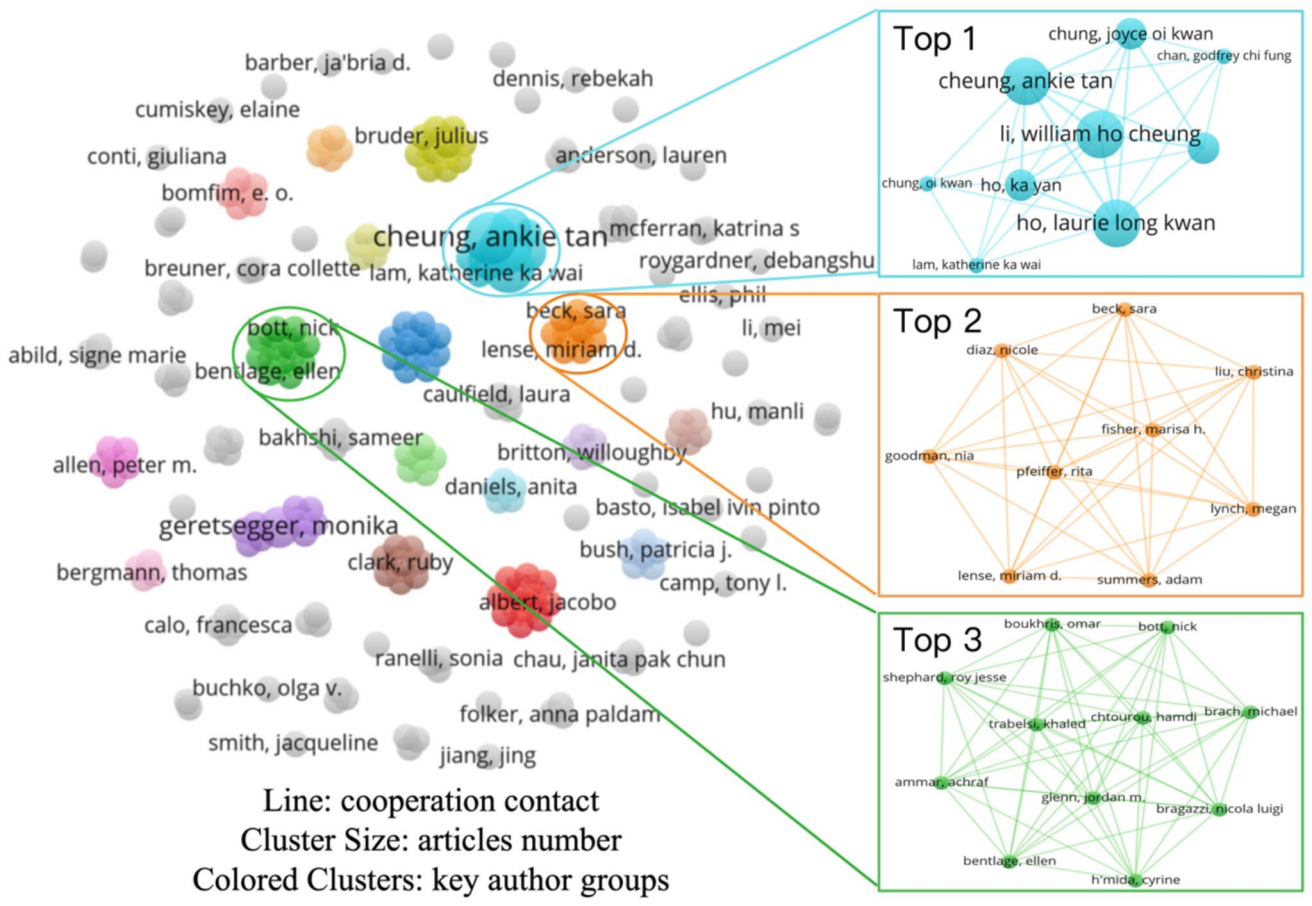
The map of author collaboration network.

Figure 3 depicts the collaboration network structure of core authors in this domain, consisting of 230 author nodes and 73 research-team clusters. Nodes are represented as circles, with size proportional to publication volume and color distinguishing different research clusters. The network layout reveals a generally dispersed author distribution, while several clusters—such as those led by Ho Cheung William Li, Sara Beck, and Nick Bott—exhibit dense interconnections, reflecting stable collaborations and sustained scholarly output.

Although overall network density remains low, the structural pattern indicates a shift from predominantly independent research toward more organized collaborative networks. The clustering of the top three research teams reveals no exceptionally large-scale research groups, thus introducing key authors with high productivity and citation rates can further explore the important research teams in this field. Although this paper selects core authors based on citation frequency, it is important to note that citation metrics may be influenced by external factors such as academic hotspots and literature dissemination channels. Future research could incorporate multi-dimensional criteria, including peer review ratings and the rigor of experimental design, to conduct a more in-depth evaluation of literature quality.

#### 3.2.2 Core authors and institutions

Table 4 presents the top five authors with ≥2 publications in this research domain and their scholarly metrics. The research team led by Ho Cheung William Li at The Chinese University of Hong Kong ranks first with three publications, accruing a total of 17 citations and an average of 5.67 citations per article. Core members of this team include Ho Cheung William Li (Associate Dean, Faculty of Medicine), Assistant Professors Ankie Tan Cheung and Laurie Long Kwan Ho. Their work focuses on pediatric and adolescent health management through the development of user-centered digital health interventions aimed at improving quality of life and psychosocial care for children with chronic illnesses, pediatric oncology patients, and adolescents from low-income families (Cheung et al., 2019, 2021; Ho et al., 2020). Associate Professor Ka Yan Ho of The Hong Kong Polytechnic University contributed as a co-author on two of these publications.

**Table 4:** Top five authors.

Rank	Author	Documents	Citations	Average citation/publication
1	Cheung, Ankie tan	3	17	5.67
2	Ho, Laurie long kwan	3	17	5.67
3	Li, William ho cheung	3	17	5.67
4	Geretsegger, Monika	2	133	66.5
5	Ho, Ka yan	2	15	7.5

Notably, Monika Geretsegger achieved the highest citation rate, with two publications cited 133 times in total (an average of 66.5 citations per article). As a clinical and health psychologist and Senior Research Fellow at the Grieg Academy Music Therapy Research Center (GAMUT), Geretsegger investigates the efficacy and applicability of music education interventions in mental health, offering in-depth analyses of key determinants of psychological well-being in children and adults (Stegemann et al., 2019; Geretsegger et al., 2022). The contributions of these prolific authors and their teams provide essential theoretical foundations and practical insights for exploring the relationship between music education and adolescents’ subjective well-being within the sustainable development framework.

#### 3.2.3 Country and regional distribution

**Figure 4:**
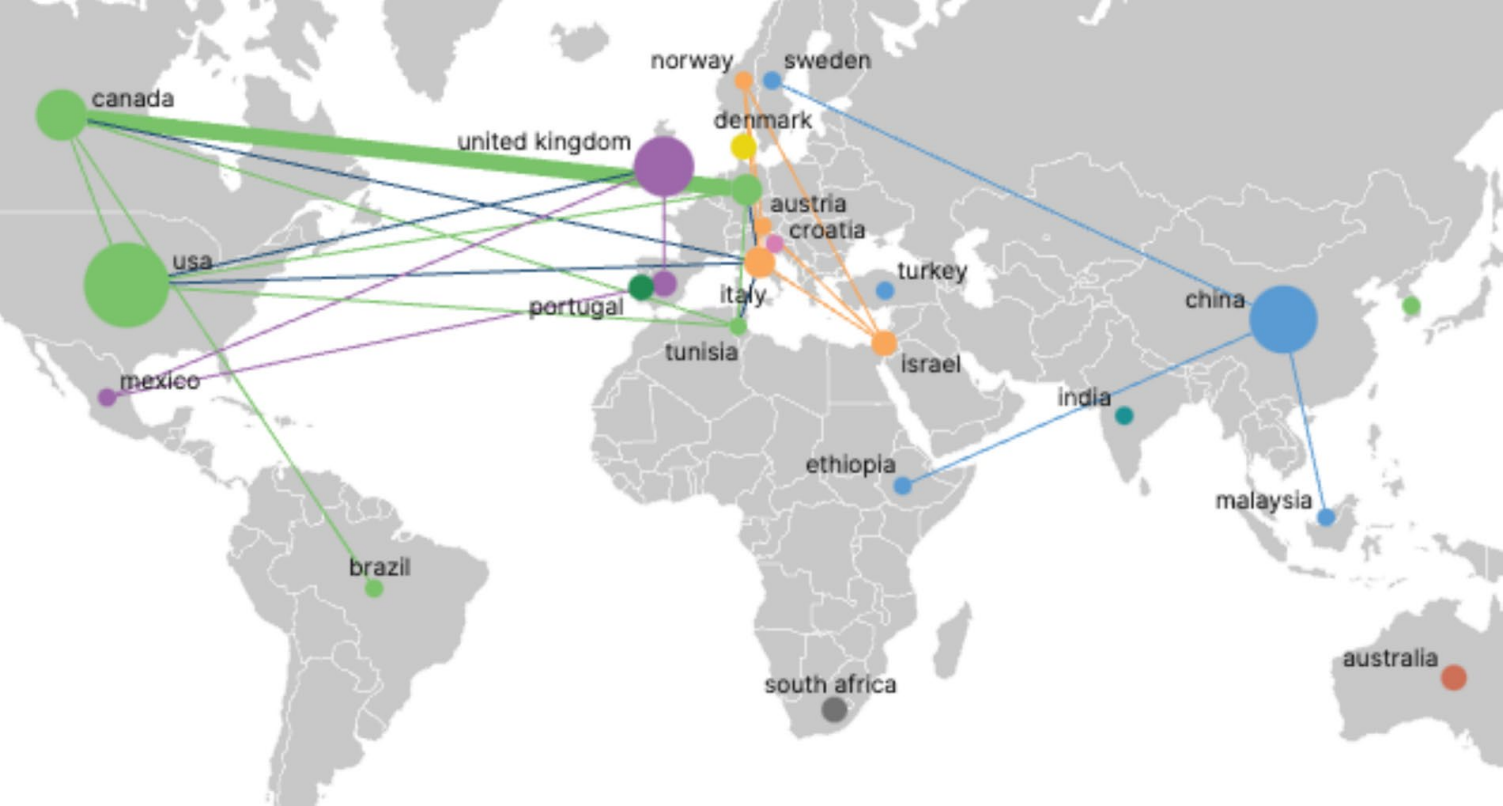
Geographic distribution map of countries.

Figure 4 presents the national cooperation network, with 24 nodes representing participating countries; node size corresponds to publication volume, and line thickness indicates the strength of inter-country collaborative output. The analysis reveals a pronounced concentration effect at the national level: a small number of core countries dominate field output. The United States, the United Kingdom, Canada, and China form a high-output cluster in North America and Europe, as evidenced by their substantially larger node sizes and the thick connecting lines denoting intensive collaboration. In contrast, most other countries exhibit lower publication counts, with smaller, more dispersed nodes and sparse connections, underscoring the geographic imbalance of research efforts and the clear predominance of North American and European contributions.

### 3.3 Content analysis (RQ3)

#### 3.3.1 Keyword clustering

**Figure 5:**
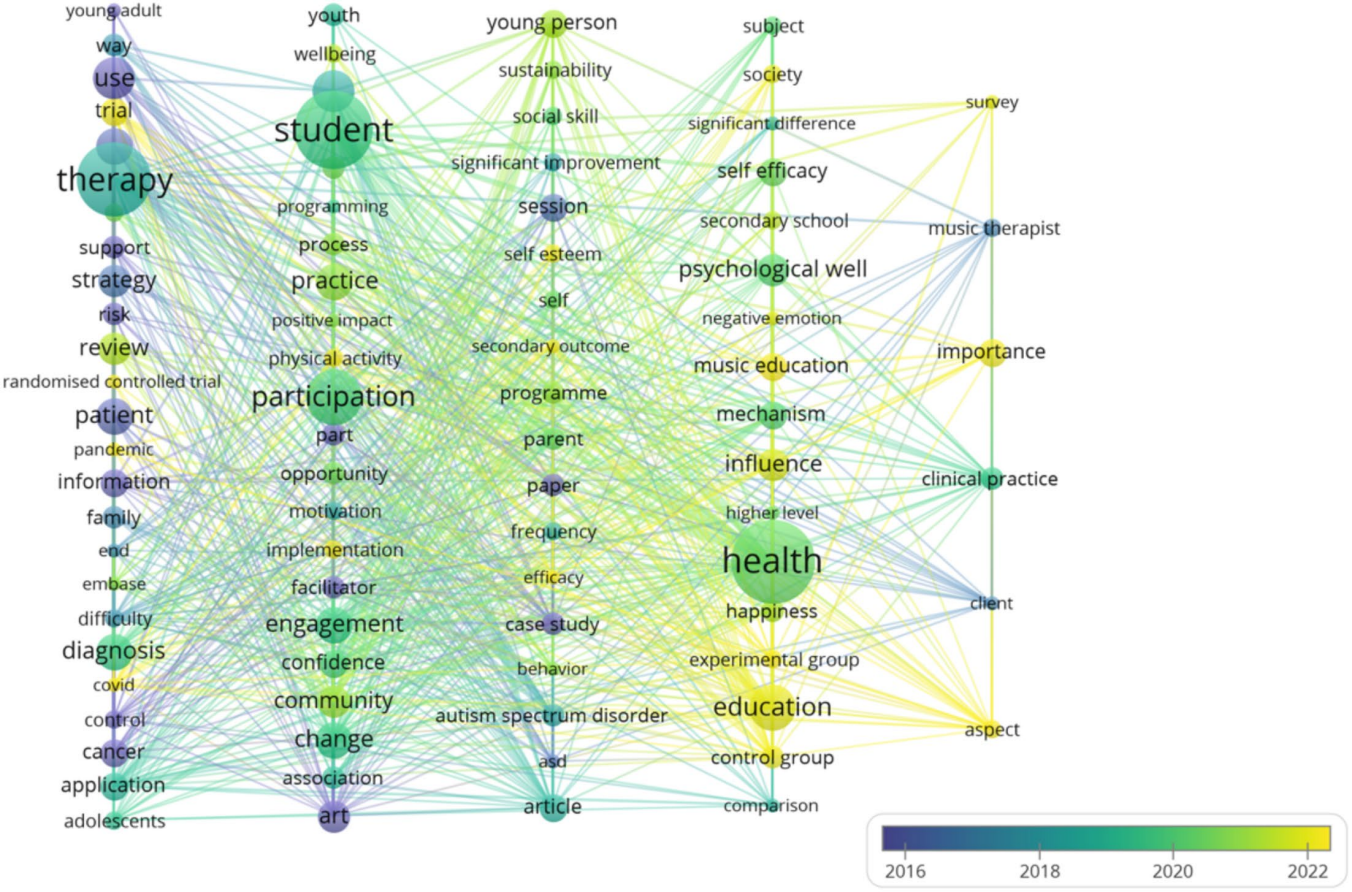
The time-zone view of keywords.

Keyword cluster analysis is an important tool for identifying hotspot topics within a research field enabling systematic capture of each hotspot’s core connotation and scope. The high-frequency keyword co-occurrence network constructed with VOSviewer (Figure 5) reveals 88 nodes aggregated into five clusters each corresponding to a distinct research theme. Specifically node size reflects keyword occurrence frequency—Larger nodes indicate higher frequency—While link thickness between nodes represents co-occurrence strength with thicker lines denoting more frequent joint appearances in the same documents; color-coded clusters delineate differentiated keyword groups clearly illustrating the distribution patterns and interrelationships of the field’s diverse research topics.

#### 3.3.2 Cluster analysis

As concise representations of research cores and academic essence, keywords—when analyzed for co-occurrence—can, through their clustering characteristics (Chen et al., 2022), outline the macro-landscape of research hotspots in a given field. This study constructs a clustering analysis framework based on keyword co-occurrence strength and semantic relevance, with visualization implemented through the probability theory-based VOSviewer software. Using the Multi-Dimensional Scaling (MDS) algorithm for dimensionality reduction of high-dimensional data and combining the Random Walk (RM) algorithm to calculate association weights between keywords, a total of 88 high-frequency keywords were ultimately classified into 5 core clusters (Table 5). Each cluster formed a research topic focus with tightly interconnected internal themes and clear external boundaries, effectively revealing the core research directions and structural characteristics of the field.

(1)  Cluster 1: Adolescent Health Interventions and Evidence-Based Practice (Red). This cluster centers on a well-established research system focusing on adolescent health interventions and evidence-based practices. Core terms such as “adolescents” and “young adult” highlight a sustained focus on intervention strategies for youth facing major health challenges (Sawni and Breuner, 2017; Chen, 2023; Kim et al., 2024). Keywords like “cancer,” “COVID,” and “pandemic” indicate intensive investigation into adolescent mental health and quality of life during public health crises (Jones et al., 2010; Satapathy et al., 2018; Chtourou et al., 2020). Methodological terms such as “systematic review,” “randomized control,” and “risk strategy” demonstrate the domain’s reliance on evidence-based medicine to develop targeted intervention tools (Lee et al., 2016; Kantor et al., 2022; Anderson et al., 2025), including digital health solutions (Bruder et al., 2021) and family support systems (Lense et al., 2020). These studies aim to address clinical challenges such as “diagnosis difficulty” (Gordon, 2022) and “treatment trial” (Carr et al., 2023), constructing comprehensive health management systems that span “patient information” and “family support” across the care continuum.
(2)  Cluster 2: Community Arts Education and Positive Youth Development (Green). Focusing on the intersection of arts education and youth social development, this cluster is anchored by terms such as “art,” “youth,” and “community” (Merati et al., 2019; Carr et al., 2023; Vazquez-Marin et al., 2023). Numerous studies affirm the benefits of music and visual arts for promoting “physical activity” (Liu, 2024), “positive impact” (Mangione et al., 2018), and “wellbeing” (Whittaker, 2015) in adolescents. Terms like “engagement,” “participation,” and “project programming” reflect how arts education initiatives operate in community settings (McFerran and Shoemark, 2013; Sarmento et al., 2024), with mechanisms such as “facilitator motivation” (Hampe, 2000) and “opportunity creation” (Park and Kim, 2024) fostering active involvement in “school–community” collaborations. The ultimate aims—“confidence building” (Fanian et al., 2015) and “social change” (Penn and Kuperberg, 2018)—highlight the role of arts education as a vehicle for constructing social capital.
(3)  Cluster 3: Behavioral Interventions for Psychological Disorders (Blue). This cluster emphasizes behavioral interventions for youth with psychological or developmental disorders, focusing particularly on “Autism Spectrum Disorder (ASD)” and “social disorders” (Walworth et al., 2009; Lense et al., 2020; Bergmann et al., 2021). Keywords like “behavior,” “social skill,” and “self-esteem” clarify the primary intervention targets (Abild et al., 2024), pursued through “case study,” “parent programme,” and “session therapy” strategies aimed at improving social interaction (Soliveres et al., 2021) and self-identity in adolescents (Cumiskey, 2022). Keywords such as “efficacy,” “significant improvement,” and “sustainability” indicate a strong emphasis on long-term outcomes (Hu, 2024), particularly in terms of relieving “secondary outcome” stress among caregivers (Lopes-Junior et al., 2016) and ensuring the “programme sustainability” of community-integrated services (Lense et al., 2020). This has led to the formation of a robust triadic model of collaboration among families, schools, and professional institutions.
(4)  Cluster 4: Educational Psychology Mechanisms and Intervention Effectiveness (Yellow). This cluster, built around keywords like “music,” “education,” and “health,” explores how music education influences adolescent psychological development (Buchko, 2020; Simunovic et al., 2022; Sulun et al., 2022). Terms such as “experimental group,” “control group,” and “significant difference” reflect the widespread use of experimental design (Kantor et al., 2022) in validating the effects of music education on indicators like “happiness” (Cheung et al., 2019), “self-efficacy” (Jiang, 2024), and “negative emotion” (Leung and Cheung, 2020). Keywords like “higher level influence” and “psychological well” suggest a growing interest in exploring deeper mechanisms—such as neurocognitive (Wang, 2023) and sociocultural processes (Kennewell et al., 2022). The cluster also highlights the differential impact of interventions across contexts like “secondary school” (Yan and Foong, 2022) and “society” (Basto, 2021), offering theory-driven support for subject-specific education policy.
(5)  Cluster 5: Clinical Practice and Professionalization of Music Therapy (Purple). As the smallest cluster, this group represents the specialized domain of music therapy’s clinical application and professionalization. Core terms such as “music therapist,” “clinical practice,” and “client” reflect the practitioner-client relationship (Dvorak, 2023), while keywords like “aspect,” “importance,” and “survey” indicate extensive research on treatment efficacy (Derrington, 2012), ethical standards (Stegemann et al., 2019), and professional demand (Dvorak, 2023). The cluster also explores the therapist’s role in emotional support (Roberts-Wolfe et al., 2012) and cognitive intervention (Li, 2024), as well as the alignment of therapy methods with clinical environments (Cheung et al., 2019), thereby laying the empirical foundation for translating practical experience into standardized intervention protocols.

**Table 5:** Cluster of keywords.

Cluster	Quantity	Keywords
Red (1)	25	adolescents, application, cancer, control, covid, diagnosis, difficulty, embase, end, family, information, pandemic, patient, randomized control, review, risk, strategy, support, systematic review, therapy, treatment, trial, use, way, young adult
Green (2)	22	art, association, change, community, confidence, engagement, facilitator, implementation, motivation, opportunity, part, participation, physical activity, positive impact, practice, process, programming, project, student, time, wellbeing, youth
Blue (3)	18	article, asd, autism spectrum disorder, behavior, case study, efficacy, frequency, paper, parent, program, secondary outcome, self, self-esteem, session, significant improvement, social skill, sustainability, young person
Yellow (4)	17	comparison, control group, education, experimental group, happiness, health, higher level, influence, mechanism, music education, negative emotion, psychological well, secondary school, self-efficacy, significant difference, society, subject
Purple (5)	6	aspect, client, clinical practice, importance, music therapist, survey

#### 3.3.3 Temporal evolution of research themes

**Figure 6:**
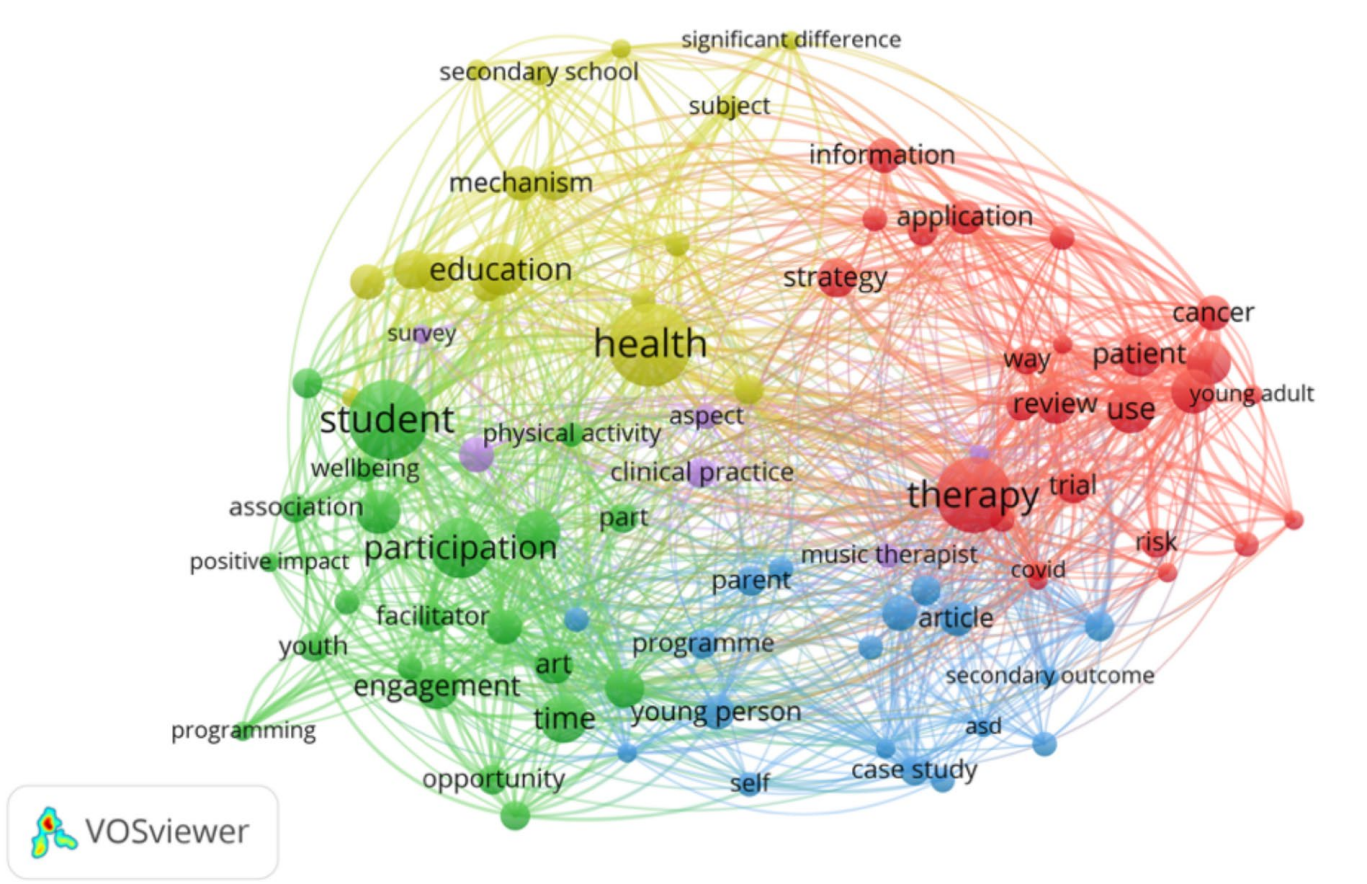
The cluster map of co-occurrence keywords.

The keyword co-occurrence clustering analysis identified five major research hotspots in the field: evidence-based interventions for adolescent health, community-based arts education, behavioral interventions for psychological disorders, educational psychology mechanisms, and clinical practice of music therapy. Based on keyword frequency and temporal distribution, 88 high-frequency terms were categorized into distinct chronological phases to trace the developmental trajectory of each research theme. As illustrated in Figure 6, the resulting three-phase keyword evolution map clearly visualizes the temporal progression of these core themes, highlighting how focus areas have shifted and expanded over time.

Based on keyword co-occurrence strength and temporal distribution, the research timeline reveals a stratified framework across three dimensions: “intervention for special populations—development of general adolescents – exploration of professional practice.”

During the Foundational Paradigm Formation Phase (2016–2018), studies primarily focused on interventions for special education populations, particularly behavioral interventions for individuals with ASD. Foundational theoretical frameworks and methodological innovations were established through scientific approaches such as systematic reviews (Kusier et al., 2024) and randomized controlled trials (Cheng et al., 2023), laying a robust methodological foundation for subsequent empirical research.

In the Practice Expansion and Deepening Phase (2019–2020), the research scope extended to general adolescent populations. Scholars emphasized mechanisms by which community-based arts education fosters adolescent social development, and how music education contributes to psychological well-being. Participatory research (Lense et al., 2020) and educational intervention programs (Good et al., 2021) served as key vehicles for embedding research findings into policy-making and educational practices.

The Responsive Professionalization Phase (2021–2022) marked a shift toward addressing pressing societal issues, such as adolescent health interventions amid the COVID-19 pandemic (Sweileh, 2020). In parallel, research advanced the standardization of professional services like music therapy, incorporating clinical practice guidelines (Derrington, 2012) and frontier technological innovations (Yang et al., 2024a) to facilitate efficient translation of research into standardized applications.

Together, these three phases outline a spiral upward trajectory—from theory-building for special needs groups, to practice-oriented pathways for general adolescent development, and onward to iterative innovation in professional services. This dynamic progression reflects the field’s sustained responsiveness to evolving societal demands and its potential for continued, sustainable advancement.

## 4 Discussion

### 4.1 Comprehensive evaluation from the perspective of sustainable development

Within the framework of the Sustainable Development Goals (SDGs), the research themes adopt a three-dimensional stratification—covering individual health, social support, and professional services—to construct an integrated evaluative system. Research on behavioral interventions for special adolescent populations highlights the necessity of establishing a coordinated “family–school–community” support mechanism, laying a sustainable foundation for their social integration (Merati et al., 2019). Studies in community arts and music education promote long-term models of individual development driven by cultural capital, enhancing youth participation and psychological resilience. These findings closely align with the SDG objective of “holistic human development” through educational practice (Shulla et al., 2020). In the domain of professional services, the standardization of music therapy—through clinical outcome evaluations and service process optimization—has fostered replicable and scalable health intervention models, offering systemic solutions to challenges such as the unequal distribution of public health resources (Barbosa et al., 2020).

The methodological practices underlying keyword clustering reflect a sustainability-oriented evaluative logic. The frequent application of evidence-based medicine in the red and blue clusters (Boso et al., 2007) ensures the scientific rigor and reproducibility of health interventions, contributing to a comprehensive health management system capable of addressing youth mental health crises and major disease challenges. The green and yellow clusters emphasize “participatory practice” and “cross-context intervention” (Abild et al., 2024), validating the long-term impact of educational environments and community-based arts programs on adolescent development. These insights offer empirical support for policymakers seeking to design sustainable educational support schemes. Research within the purple cluster, focused on clinical practice and professional norms (Melesse et al., 2022), promotes a shift in the field of music therapy from experience-driven to evidence-driven practice, fostering a sustainable ecosystem for professional development through clear role definition and service standardization.

From a temporal perspective, the evolution of research themes demonstrates dynamic alignment with societal needs, embodying a model of sustainable development in motion. Early-stage methodological frameworks have equipped mid-stage practice expansions with robust theoretical tools, which in turn informed later research shifts toward public health responses. This dynamic adaptability manifests not only in the deepening of individual research domains, but also through synergistic effects across clusters. Co-occurring terms such as “well-being” and “youth” link diverse objectives spanning health (Stegemann et al., 2019), education (Boyce-Tillman, 2000), and social support (Cheung et al., 2021). Collectively, these research streams—through continued empirical accumulation, optimized intervention pathways, and strengthened professional standards—form a sustainable development research paradigm equipped to address complex societal challenges. This paradigm provides both scientific and practical evaluative frameworks for ensuring the long-term well-being of adolescent populations (Chase-Lansdale et al., 2011).

### 4.2 Suggestions on future sustainable music education issues

A multidimensional music education practice system rooted in “contextual integration and needs orientation” should be established. The sustainable development of future music education requires a departure from the traditional classroom model, developing an integrated practice network that encompasses both school and community settings. The green cluster (Cluster 2) takes “community arts education” as the core and systematically demonstrates the unique value of music education to the social development of adolescents outside of school settings (Merati et al., 2019; Carr et al., 2023; Vazquez-Marin et al., 2023). Within schools, the deep integration of music education with mental health education should be strengthened (Mirović and Bogunović, 2013). Through curriculum design, music education interventions can be embedded into developmental goals such as emotional regulation and social skills training for adolescents, forming a normalized mechanism of psychological support. At the community level, mature models such as “arts education for positive youth development” (Liu, 2023) may serve as a foundation for music workshops and intergenerational music projects centered on youth participation. These initiatives can leverage music’s cultural connectivity to enhance community cohesion. For special youth populations, it is essential to further optimize the coordination between music therapy and behavioral interventions. Personalized music strategies should be explored in domains such as ASD to ensure that all groups with diverse needs can access sustainable support through music education (Low et al., 2024).

An innovative paradigm of music education driven by “technological empowerment and evidence-based practice” must be promoted. In the context of digital transformation, sustainable music education development requires the integration of technological innovation with empirical research. The red cluster (Cluster 1) explicitly mentions the application of digital health solutions (Bruder et al., 2021) and family support systems (Lense et al., 2020) in adolescent health interventions, and emphasizes the use of clinical practices such as “diagnosis difficulty” (Gordon, 2022) and “treatment trial” (Carr et al., 2023) to verify the intervention effects, providing methodological support for the idea that “technology applications must be based on empirical evidence.” On one hand, immersive music learning tools—enabled by technologies such as artificial intelligence and virtual reality (Cross, 2023)—can address challenges like limited teaching resources in remote areas and imbalanced resource distribution, establishing scalable hybrid (online-offline) educational models. On the other hand, the methodological strengths of prior research—such as controlled experiments and evidence-based practice—should be continued to systematically evaluate the long-term effects of music education on outcomes like adolescent self-efficacy and social adaptation (Root Wilson, 2009), with particular attention to sustained mechanisms for mitigating negative emotional states following public health crises. This transition from experience-based to data-driven practice will enhance the scientific positioning and practical value of music education in the modern educational system.

A comprehensive support network for music education should be advanced through “professional collaboration and ecological co-construction.” The blue cluster (Cluster 3) and the purple cluster (Cluster 5) indicate that sustainable development of music education depends on cross-disciplinary collaboration and professional ecological construction. Core keywords such as special youth education (Walworth et al., 2009; Lense et al., 2020; Bergmann et al., 2021), ethical norms (Stegemann et al., 2019) and industry needs (Dvorak, 2023) highlight the need for ecological construction of music therapy as an independent professional field. In terms of teacher training, it is critical to integrate resources from disciplines such as musicology, psychology, and special education, thereby constructing a training system for interdisciplinary professionals (Karkina et al., 2021) and strengthening teachers’ capacity to adapt to diverse settings such as mental health and community education. On the policy level, music education should be integrated into macro-level planning for youth mental health services and public cultural development (Aitchison and McFerran, 2022), with interdepartmental cooperation mechanisms established to enable resource integration and coordinated intervention. In research, deeper exploration of topics such as the “mechanisms of music education impact” (Jaschke et al., 2013) and “standardization of clinical practices for music therapists” (Waldon, 2016) should be encouraged. This will bridge the gap between fundamental theory, applied research, and policy translation, providing systemic support for the long-term role of music education in promoting adolescents’ holistic development.

### 4.3 Limitations and future directions

Although this study constructs a clear research landscape through a standardized literature review methodology, its methodological framework still presents room for improvement due to limitations in cross-dimensional integration and the analysis of complex mechanisms. First, the structured retrieval strategy based on the PICO framework may introduce selection bias against emerging studies that have yet to establish clearly defined intervention–control structures, thus potentially constraining the capture of frontier topics. Second, while keyword co-occurrence analysis effectively reveals the internal mechanisms of the research field, it fails to fully address deeper patterns such as mediation effects, moderating roles, and outcome differentiation. Third, the study lacks adequate integration of qualitative data—such as teacher interviews and adolescent narratives—which could further enrich the contextual adaptation mechanisms of specific intervention pathways.

Future research should pursue breakthroughs in both methodological systems and research dimensions. First, it is recommended to adopt a mixed-methods paradigm that combines qualitative approaches—such as grounded theory and case tracking—with quantitative metrics. This would enable in-depth analysis of the dynamic processes through which music education influences adolescent well-being, with special attention to the unique experiences and differentiated needs of marginalized groups such as youth with disabilities and children of migrant workers. Second, research should extend its temporal and spatial dimensions. Longitudinal data collection across multiple years would allow for the evaluation of long-term impacts of music education interventions, with a particular focus on sustained outcomes in adult social adaptability and mental health. This approach would support the construction of a full-cycle evaluation model encompassing “short-term effects – medium-term development – long-term well-being.” Third, interdisciplinary collaborative research should be strengthened by integrating theoretical tools from neuroscience, sociology, and public policy. This would enable exploration of innovative applications of music education in promoting social equity and responding to global crises. Future research can thus consolidate the scientific foundation for “music education as a driver of sustainable adolescent development” and provide more targeted frameworks for policy formulation and practical innovation.

## 5 Conclusion

This study, grounded in the perspective of sustainable education, explores the impact of music education on adolescents’ subjective well-being through a systematic literature review, addressing three core research questions in depth. From a temporal perspective, the field has evolved from a preliminary theoretical phase before 2018 into a period of rapid growth thereafter, with the surge in research activity closely aligned with the advancement of global Sustainable Development Goals. Spatial analysis indicates the emergence of collaborative networks among key researchers—such as the team led by Ho Cheung William LI at the Chinese University of Hong Kong and the group led by Monika Geretsegger at the Grieg Academy—yet the field remains predominantly concentrated in North America and Europe, reflecting a significant geographical imbalance. In terms of content, high-frequency keyword co-occurrence analysis identifies five major research clusters: “evidence-based interventions for adolescent health,” “community arts education facilitation,” “behavioral interventions for psychological disorders,” “educational psychological mechanism exploration,” and “clinical practice of music therapy.” These clusters follow a three-stage spiral trajectory: the construction of theoretical frameworks for special populations (2016–2018), practical expansion to general adolescent development (2019–2020), and the standardization of professional public health services (2021–2022).

Within the framework of the SDGs, this study establishes a multidimensional evaluation system encompassing individual health, social support, and professional services. Methodologically, the study rigorously adheres to the PICO framework and PRISMA guidelines, constructing a standardized, replicable systematic review model to ensure reliability and scientific rigor. Visualization tools such as VOSviewer are employed to translate research hotspots into quantifiable co-occurrence networks, offering innovative methodologies and perspectives for interdisciplinary inquiry. Theoretically, the study systematically reveals the diverse mechanisms through which music education enhances adolescent well-being, enriching the theoretical chain of “educational intervention—psychological development—sustainable well-being,” and expanding the role of quality education in advancing human development. On the practical level, the discussion and suggestions of the study provide valuable references for policymakers. Whether it is building a three-dimensional practice network, promoting technology-enabled intervention models, or establishing cross-departmental cooperation mechanisms, it provides a practical direction for music education to play a greater role in the comprehensive development of young people.
